# Protocol for identifying head direction cells in mice using *in vivo* extracellular recordings and juxtacellular labeling

**DOI:** 10.1016/j.xpro.2026.104836

**Published:** 2026-09-16

**Authors:** Shan Jiang, Francesca Corsi, Sara Hijazi, Tim J. Viney

**Affiliations:** 1 Department of Pharmacology, University of Oxford, Oxford, UK; 2 Department of Pharmacy, University of Pisa, Pisa, Italy

**Keywords:** Single Cell, Neuroscience, Systems biology

## Abstract

Head direction (HD) cells are required for spatial orientation. Protocols that combine functional identification with anatomical recovery of single HD neurons remain technically demanding. We present a protocol for recording HD cells in awake, head-fixed mice using *in vivo* extracellular single-neuron recordings during passive rotation. We describe steps for craniotomy, habituation, glass-electrode recordings, and online identification of HD cells. We then detail procedures for juxtacellular labeling with neurobiotin, high-density probe recordings, and *post hoc* histological recovery.

For complete details on the use and execution of this protocol, refer to Hijazi et al.^1^ and Jiang et al.^2^

## Highlights

• Online identification of head direction cells in head-restrained mice
• Extracellular recording and juxtacellular labeling of HD cells
• *Post hoc* recovery of soma, dendrites, and axons of recorded HD cells
• Neurochemical profiling of recorded HD cells

## Abstract (editor highlights)

Publisher’s note: Undertaking any experimental protocol requires adherence to local institutional guidelines for laboratory safety and ethics.

## Graphical abstract

**Figure:**
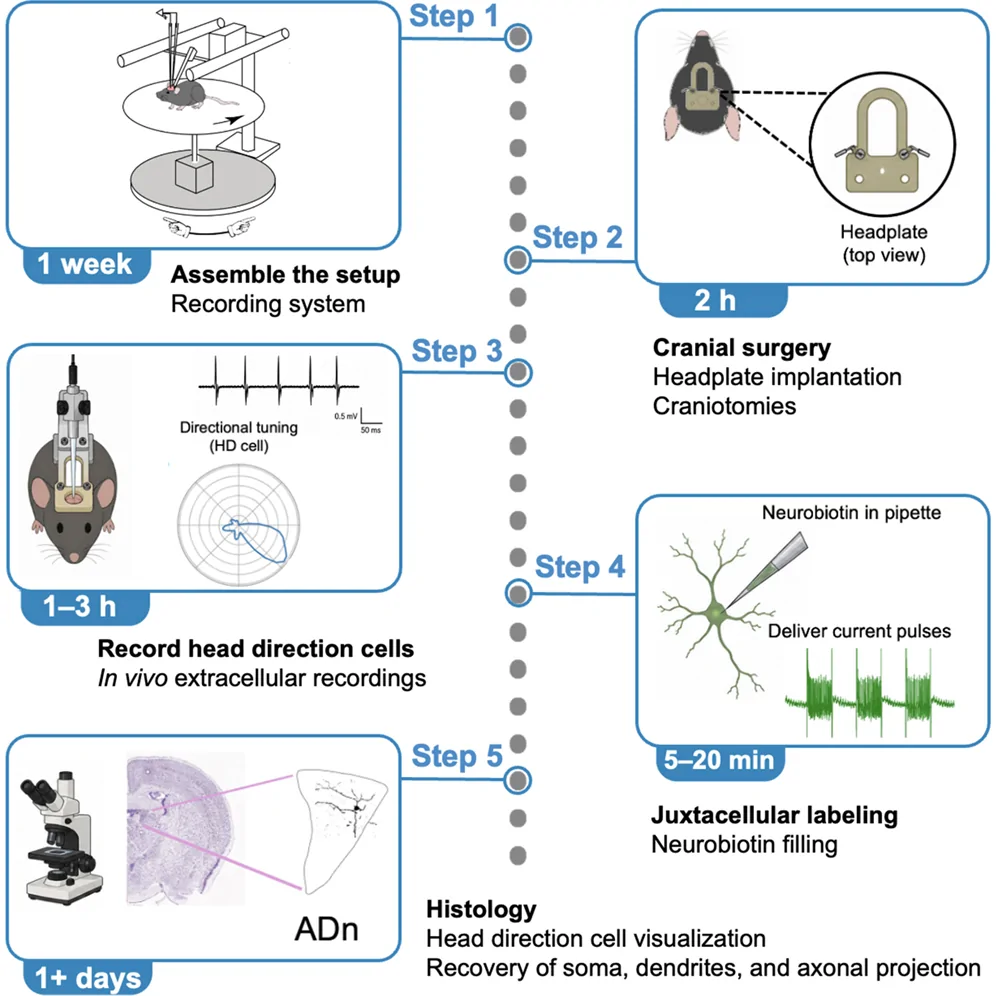

## Before you begin

Head direction (HD) cells fire maximally when an animal faces a specific direction. At the population level, HD cells provide a neural representation of an animal’s orientation, which comprises a core component of the mammalian spatial navigation system.^3^ Linking HD activity to the neurochemical profile and connectivity of individually recorded neurons is important for defining cell types and circuits responsible for directional coding. This protocol describes a workflow for accessing HD cells in drug-free awake mice by combining *in vivo* extracellular single-neuron recordings with juxtacellular labeling,^4^^,^^5^^,^^6^ followed by *post hoc* recovery and identification of recorded neurons.^1^^,^^2^^,^^7^ We first outline the assembly of a passive-rotation setup for detecting and recording HD cells. We then describe cranial surgical steps for performing headplate implantation and craniotomies, followed by blind glass-electrode recordings, online identification of HD cells during passive rotation, and juxtacellular labeling of recorded neurons with neurobiotin. This is followed by optional targeted recordings with silicon probes. Finally, we detail procedures for visualization of neurobiotin-labeled cell processes. Together, these procedures enable direct structure-function mapping of anatomically identified HD cells in the anterodorsal thalamic nucleus (ADn).

### Innovation

This protocol advances existing approaches by integrating four elements into a single reproducible workflow: stable *in vivo* extracellular single-cell recordings in awake head-fixed animals, online functional identification of HD cells during passive rotation, accurate silicon probe placement for targeted population-level recordings, and recovery of juxtacellularly-labeled HD cells for *post hoc* anatomical analysis. Passive rotation facilitates mechanical stability while preserving robust HD tuning, enables flexible recording conditions (e.g., maintaining a cell within or outside the preferred firing direction, presenting sensorimotor stimuli), allows repeated acquisition across the full angular range, and increases the success rate for juxtacellular labeling compared to freely-moving conditions.

A further advantage is that the protocol supports direct comparison of physiological properties, such as HD parameters, spike shape, and firing patterns, with cell position, cell shape, and axon projection pattern. In addition, the recovered juxtacellularly labeled neurons can be combined with immunohistochemistry.^1^ This enables investigators to test the neurochemical identity of anatomically and physiologically defined HD cells and to relate directional coding to cell-type-specific markers. Because cell types are typically defined by convergent anatomical, neurochemical, and electrophysiological features, this combined workflow provides a practical way to assign molecular identity to recorded HD cells and to compare firing properties across defined neuronal classes.

The workflow is also flexible and can be adapted for other brain regions in the HD network, such as the postsubiculum.^8^^,^^9^^,^^10^^,^^11^ Overall, this protocol provides a practical route to map directional coding properties to cell type and circuit identity.

### Institutional permissions

All animal procedures were approved by the local Animal Welfare and Ethical Review Body under approved personal and project licenses in accordance with the Animals (Scientific Procedures) Act, 1986 (UK) and associated regulations.

### Preparation of the *in vivo* recording setup

**Timing: 1 week**

**Figure 1:**
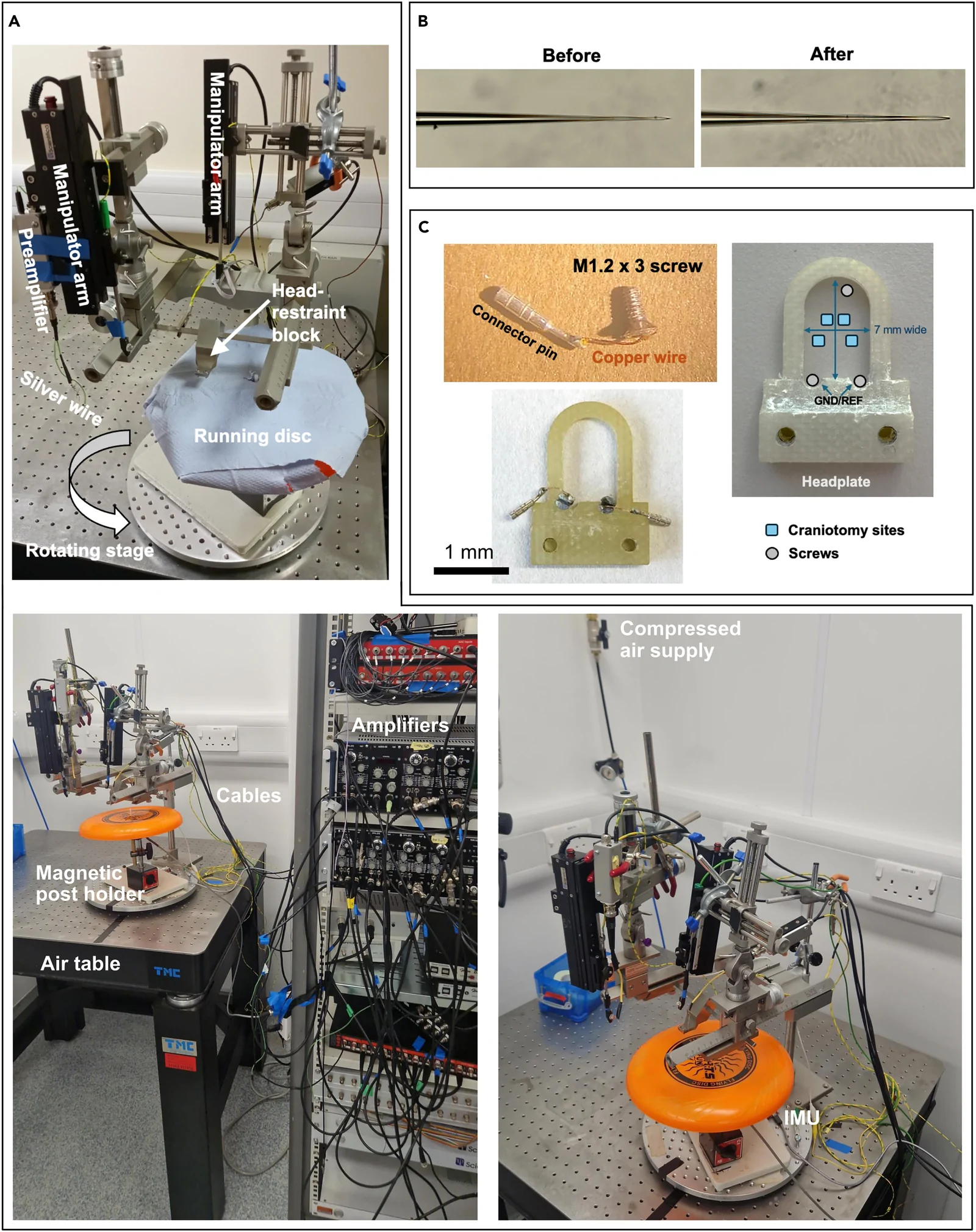
Recording setup assembly, glass pipettes, and custom headplate components (A) Recording setup used for *in vivo* extracellular recordings in awake head-fixed mice (three different views are shown). The setup includes a circular turntable with a running disc, two micromanipulator arms for positioning glass electrodes, preamplifiers mounted to the manipulator arms, and a custom head-restraint block mounted onto a heavy-duty frame. Silver wires are connected to preamplifiers via trimmed BNC cables. The inertial measurement unit (IMU) is attached to the turntable behind the magnetic post holder. (B) Representative glass pipette tips before and after polishing. Pipettes are pulled from borosilicate glass capillaries and polished to obtain suitable tip geometry and electrode impedance for extracellular recordings and juxtacellular labeling. The final tip diameter is ∼1 μm.^5^ (C) Components used for headplate implantation and grounding. Top left: copper wire soldered to a female connector pin and a M1.2×3 screw. Bottom left: custom lightweight headplate used for head restraint. Right: schematic representation of headplate positioning showing craniotomy sites and skull screw locations used for ground/reference connections and support (headplate anchor points).
2. Assemble electrophysiological recording interface.
  a. Chloride several silver wires by immersing them in sodium hypochlorite solution until the surface of the silver wire turns dark gray (∼4 hours), which improves electrical stability and reduces noise.
  b. Solder each silver wire to a gold pin.
  c. Connect a silver wire via the gold pin to a trimmed BNC cable and connect this to the preamplifiers mounted to the manipulator arms (Figure 1A).
  d. Connect the preamplifier to amplifier modules via long BNC cables that can be trailed behind the heavy-duty frame on a three-prong clamp (important for balancing the recording setup for passive rotation).
  e. Connect amplifiers to an analog-to-digital converter (ADC).
    ***Note:*** Wideband signals (local field potentials, LFPs) are acquired with DC coupling, 0.1 Hz–8 kHz, 1000× gain. Filtered action potentials are acquired in parallel with AC coupling, 0.4–8 kHz, 1000× gain. ‘Hum Bugs’ are recommended for the removal of mains or line noise.
  f. Connect the ADC to a computer installed with appropriate data acquisition software (e.g., Spike2).
    i. Digitize samples at 20 kHz.
  g. Connect the IMU to the computer and install appropriate software (Arduino IDE).
3. Prepare glass electrodes for *in vivo* single-cell extracellular recordings.
  a. Use a gravity micropipette puller and glass capillaries (1.2 mm outer diameter, 0.69 mm inner diameter).
    i. Optimize settings for the heat, main magnet, and sub-magnet.
  b. Pull pipettes to produce a tip diameter of ∼0.5–1 μm.^5^
  c. Under a microscope (20× magnification), polish the tip of each pipette using a glass rod.
  d. Verify the shape of the pipette tip with 40× magnification (Figure 1B).

***Note:*** The optimal tip should be flat, smooth, and refract blue light.

1. Assemble the recording rig (Figure 1A).
  a. Mount a heavy-duty frame to a turntable on an air table, enabling 360° passive rotation.
  b. Install a lightweight 25 cm diameter plastic running disc covered with paper roll underneath the head-restraint device on a brass post (connected via a nylon bushing from a standard mouse running wheel).
    ***Note:*** This design facilitates spontaneous running and rest periods during head-restraint. The height of the running disc relative to the head-restraint block can be adjusted for each mouse if required.
    ***Note:*** The optimal distance between the center of running disc and the mouse’s head is ∼7 cm.
  c. Attach a rotary encoder to the post underneath the running disc to monitor the animal’s running speed during *in vivo* recordings.
  d. Fix an inertial measurement unit (IMU) to the turntable to enable monitoring of direction.
  e. Assemble two ultra-stable low-noise robotic micro-manipulators on standard manipulator arms to control the position of the two glass electrodes.

4. Prepare aliquots of filtered neurobiotin solution (3% in 0.5 M sodium chloride) and store them at −20 °C.

### Prepare for surgical procedures

**Timing: 1 h**

5. Autoclave surgical tools: skin hooks, scissors, coarse and fine forceps, cotton buds, swabs.
6. Prepare ground/reference screws by soldering M1.2×3 machine screws to female connector pins using copper wires (Figure 1C). Two required per mouse; it is recommended to prepare several spares.
7. Prepare items for cold sterilization: headplate, screws.
8. Check status of equipment, drugs, and consumables (including stereotaxic frame, surgical microscope, micromanipulator, buprenorphine, iodine/chlorhexidine, saline, screwdriver, dental cement).
9. Habituate mouse to the recording room and recording setup prior to surgical procedures.

***Optional:*** An additional coiled steel wire can be prepared by soldering it to a female pin to enable electromyography (EMG) for monitoring muscle tone and behavioral states during *in vivo* recordings.^1^

## Key resources table

**Table:** 

REAGENT or RESOURCE	SOURCE	IDENTIFIER
**Chemicals, peptides, and recombinant proteins**		

Neurobiotin tracer	Vector Laboratories	SP-1120; RRID: AB_2313575
Alexa Fluor 488-, Cy3- or Cy5-conjugated streptavidin	Jackson ImmunoResearch	Lot# 151925, 37442
Isoflurane inhalation anesthesia (IsoFlo)	Teva Ltd. Zoetis	https://products.tevauk.com/p/Category?id=185
Vetergesic (buprenorphine), 0.1 mg/kg dose	Ceva	56454-04
Marcain (bupivacaine)	Aspen	PS22421
Metacam (meloxicam), 5 mg/kg dose	Boerhinger Ingelheim	N/A
Videne (Iodinated povidone)	ECOLAB	3030440
Refobacin bone cement	Zimmer Biomet	3003940002-3
Tetric EvoFlow dental cement	Ivoclar Vivadent	A1
Viscotears Liquid Gel (carbomer 0.2%)	Novartis	N/A
EMLA cream (lidocaine 2.5%/prilocaine 2.5%)	AstraZeneca	N/A
DiI tracer	Life Technologies	V22889

**Critical commercial assays**		

Vectashield Antifade Mounting Medium	Vector Laboratories	H-1000; RRID: AB_2336789
Vectastain Elite ABC-HRP Kit	Vector Laboratories	PK-6200

**Experimental models: Organisms/strains**		

C57BL6j mice (adult, male and female)	Charles River Laboratories	https://www.criver.com/

**Software and algorithms**		

MATLAB	https://www.mathworks.com/products/matlab.html	RRID:SCR_001622
Python	https://www.python.org/	RRID:SCR_008394
ZEN software	https://www.zeiss.com/microscopy/en/products/software/zeiss-zen.html	RRID:SCR_018163 RRID:SCR_013672
Spike2	http://ced.co.uk/	RRID:SCR_000903
Arduino IDE	https://www.arduino.cc/en/software/	RRID:SCR_024884
Phy	https://github.com/cortex-lab/phy	N/A
LinLab 2	https://www.scientifica.uk.com/products/scientifica-linlab-2	N/A

**Other**		

CED Power1401 analog-to-digital converter	http://ced.co.uk/products/power3	RRID:SCR_016040
Scientifica IVM-1000 single stereotaxic motorized manipulator	https://www.scientifica.uk.com/products/scientifica-ivm-single	RRID:SCR_021035
Amplifier modules	https://www.npielectronic.com/	ELC-01MX, DPA-2FS
Timer and audio modules	https://www.npielectronic.com/	TMR-02M, AUDIS-03
Hum Bug Noise Eliminator	https://www.digitimer.com/	N/A
Micropipette puller	NARISHIGE scientific instrument lab	PE-21
Glass capillaries (pipettes)	Harvard apparatus	GC120F-10
Flexible needle	World Precision Instruments	Microfil 34 Gauge/76 mm length, MF34G-5
Disposable sterile scalpels	Swann-Morton	N/A
Sugi Eyespear pointed tip	KETTENBACH Simply intelligent	REF 30601
Sterile drapes	KRUUSE	BUSTER
Drill bits	Fine Science Tools	19007-05; 19007-07
1 ml syringes	HENKE-JECT	5010-A00V0
30G needles	TERUMO	AN∗3013R1
Isoflurane anesthesia system	AW Anaesthesia services Ltd.	N/A
Homeothermic monitoring system	Harvard Apparatus	55-7020
Hair clipper	WAHL	N/A
Heavy-duty frame	Kopf Instruments	Model 1430
Headplates	Custom-made	Machined glass-reinforced plastic headplate
Head-restraint block	Custom-made	Steel block adapted to fit heavy-duty frame
Circular breadboard	Thorlabs, Inc.	MBR12U
Stereotaxic apparatus	Kopf Instruments	Model 963
Non-rupture ear bars	Kopf Instruments	1922
Surgical microscope	Zeiss	OPMI pico Vario 100
Machine screws	Precision technology supplies	A96301202, A96301203
Vibrating microtome	Leica Microsystems	VT 1000S vibratome
Silicon	Smooth-On	Body Double
Inertial measurement unit	SparkFun	OpenLog Artemis
Silver wires	A-M Systems	782500

## Step-by-step method details

### Headplate implantation and craniotomies

**Timing: 2 h**

1. Prepare for surgical procedures.
  a. Establish sterile working area (disinfect surgical area including microscope and frame).
  b. Weigh mouse.
  c. Turn on homeothermic monitoring system.
  d. Anesthetize the mouse using isoflurane (∼4% in carboxygen, 2 L/min flow rate) inside an induction chamber.
  e. Clip hair with clippers before transferring the anaesthetized mouse to the nose cone on the stereotaxic frame.
  f. Inject buprenorphine (0.1 mg/kg) subcutaneously; isoflurane can be maintained at 1–2% for the duration of the surgery.
  g. Place mouse in jaw bar and carefully fix non-rupture ear bars without damaging ear canals.
  h. Secure rectal probe and maintain body temperature at 35.6–37.5°C.
  i. Apply ocular lubricant.

**CRITICAL:** Throughout the surgery, regularly check body temperature, respiratory rate, and that eyes remain lubricated.

2. Begin aseptic phase of surgery.
  a. Disinfect scalp with chlorhexidine/iodine and administer a subcutaneous injection of bupivacaine.
  b. Make a single incision along the midline of the scalp using a sterile scalpel blade.
  c. Use sterile skin hooks to retract scalp and expose cranium, including the interparietal bone.
  d. Retract the muscle attachments from lateral ridges near the lambdoid suture (this reduces movement-induced artifacts during *in vivo* recordings).
  e. Carefully scrape the periosteum and score skull with scalpel (important for bone cement adherence).
  f. Using a stereotaxic arm and glass pipette, ensure Bregma and Lambda reference points are aligned.^12^
  g. Zero coordinates at Bregma and mark positions of craniotomy sites.
    ***Note:*** For adult C57Bl6j mice, recommended coordinates from Bregma for the ADn are: antero-posterior (AP) −0.80 mm, medial-lateral (ML) ±0.75 mm. For CA1: AP –2.50 mm, ML ±1.50 mm. (Figure 2A)
    **Figure 2:**
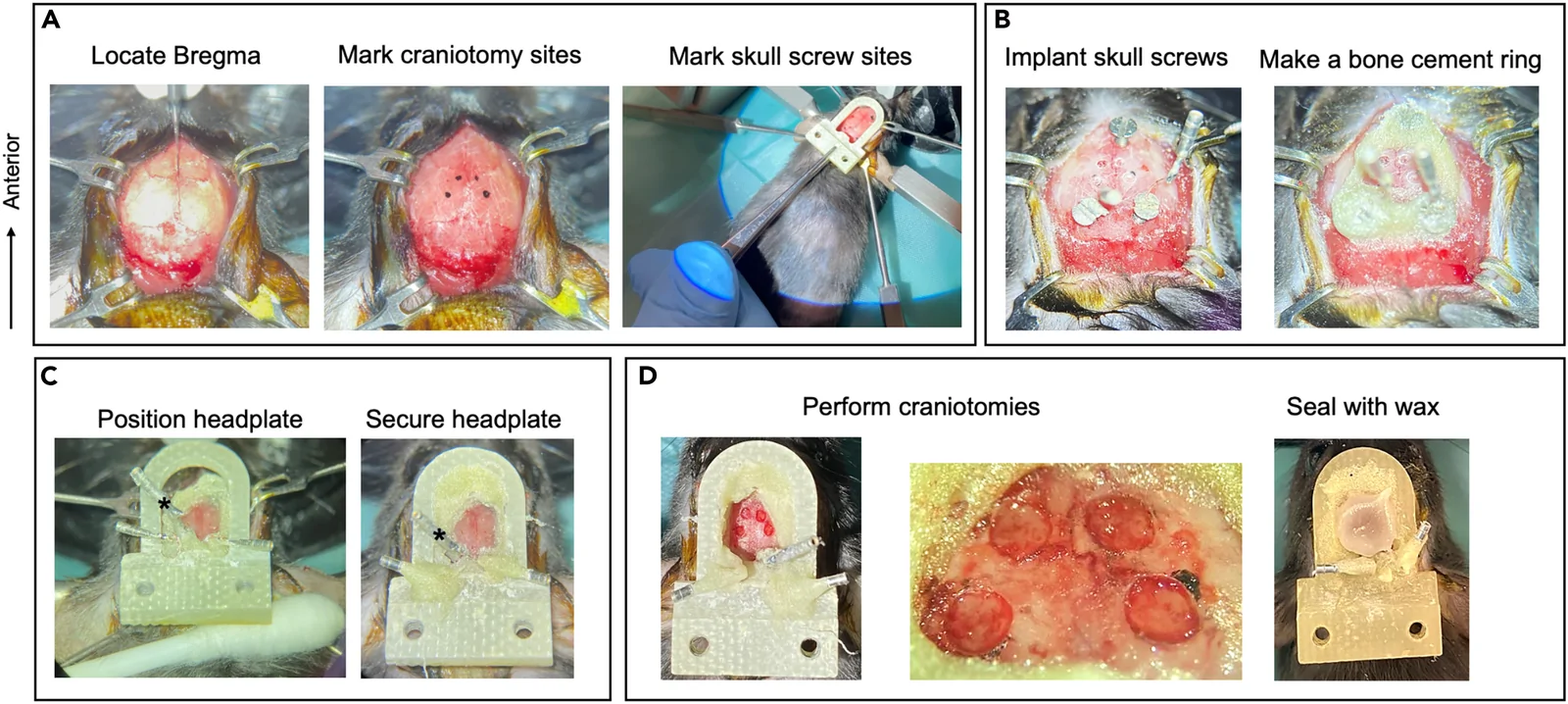
Surgical workflow for headplate implantation and craniotomy (A) Identification of Bregma and marking of craniotomy sites and skull screw locations. (B) Implantation of skull screws and application of bone cement. (C) Positioning and fixation of the headplate. Two pins are exposed that connect to the screws in the interparietal bone (ground/reference). Optional: include a third pin (asterisk) connected to a coiled steel wire positioned in the neck muscle (for EMG). (D) Left, drilling of craniotomies above recording target regions. Middle, example of completed craniotomies. Right, protection of cranium with wax after the surgery.
  h. Partially drill burr holes at marked sites with a surgical microdrill.
  i. Temporarily position the headplate over the cranium and mark with a pen the sites for skull screw anchor points, which includes one on the frontal bone and two on the interparietal bone (which also become ground/reference sites). (Figure 2A)
  j. Drill burr holes at the marked sites, without damaging the dura.
  k. Secure the two M1.2×3 screws (with soldered female pins) to the sites on the interparietal bone and secure one or two M1.2×2 screws on the frontal bone (+1.5 AP, ±1.7 ML). (Figure 2B)
  l. Seal screws with bone cement.
  m. Make a bone cement ring around the screws and marked craniotomy sites (Figure 2B).
  n. Attach headplate over the screws and align with the surface of the skull. Secure with bone cement (Figure 2C).
    ***Note:*** Place a cotton bud underneath the headplate to keep the headplate at the correct level while the bone cement dries.
    ***Optional:*** Implant a coiled steel wire into the neck muscle for an EMG contact (Figure 2C).
3. Perform craniotomies.
  a. Use a small diameter drill bit to perform craniotomies. Ensure that edges are straight. (Figure 2D)
  b. Control any bleeding with absorbable sponges.
  c. Carefully remove bone flaps from each site using a bent sterile needle as a hook.
  d. Pierce dura and gently remove dura with the needle.
  e. Rinse with sterile saline.

**CRITICAL:** All bone flaps must be removed to reduce the chances of inflammation and ensuring glass pipettes do not break when establishing *in vivo* recordings.

4. Post-operative recovery.
  a. Cover craniotomy sites with silicon or warm paraffin wax (Figure 2D).
  b. Administer post-operative analgesic (e.g., meloxicam at 5 mg/kg).
  c. Place the animal in a cage over a pre-heated heat blanket.
  d. Monitor the animal continuously until recovery of consciousness.

### Habituation to head fixation and passive rotation

**Timing: 1–3 days**

5. Place the animal on the running disc for 5 min before securing the headplate via the head-restraint block.
6. Habituate the animal gradually, ensuring that the mouse is able to run and rest on the running disc.
7. Perform passive rotations during head-restraint (1 degree/s).

***Note:*** Continue habituation until the animal remains calm and exhibits spontaneous running and resting periods on the running disc during passive rotation. Conduct one to three habituation sessions per day. Increase the duration of head-restraint by 10 to 20 mins across sessions. Rewards are not required. Maintain a cue-rich environment with stable distal visual landmarks, which help anchor HD cells.

### *In vivo* extracellular recordings of HD cells

**Timing: 1–3 h**

***Note:*** This section describes the complete procedures for each recording session, which can be repeated across multiple days as long as the craniotomy sites are accessible and the glass pipette penetrates successfully. At the end of each recording session, apply warm wax to protect the craniotomy sites (Figure 2D).

**Figure 3:**
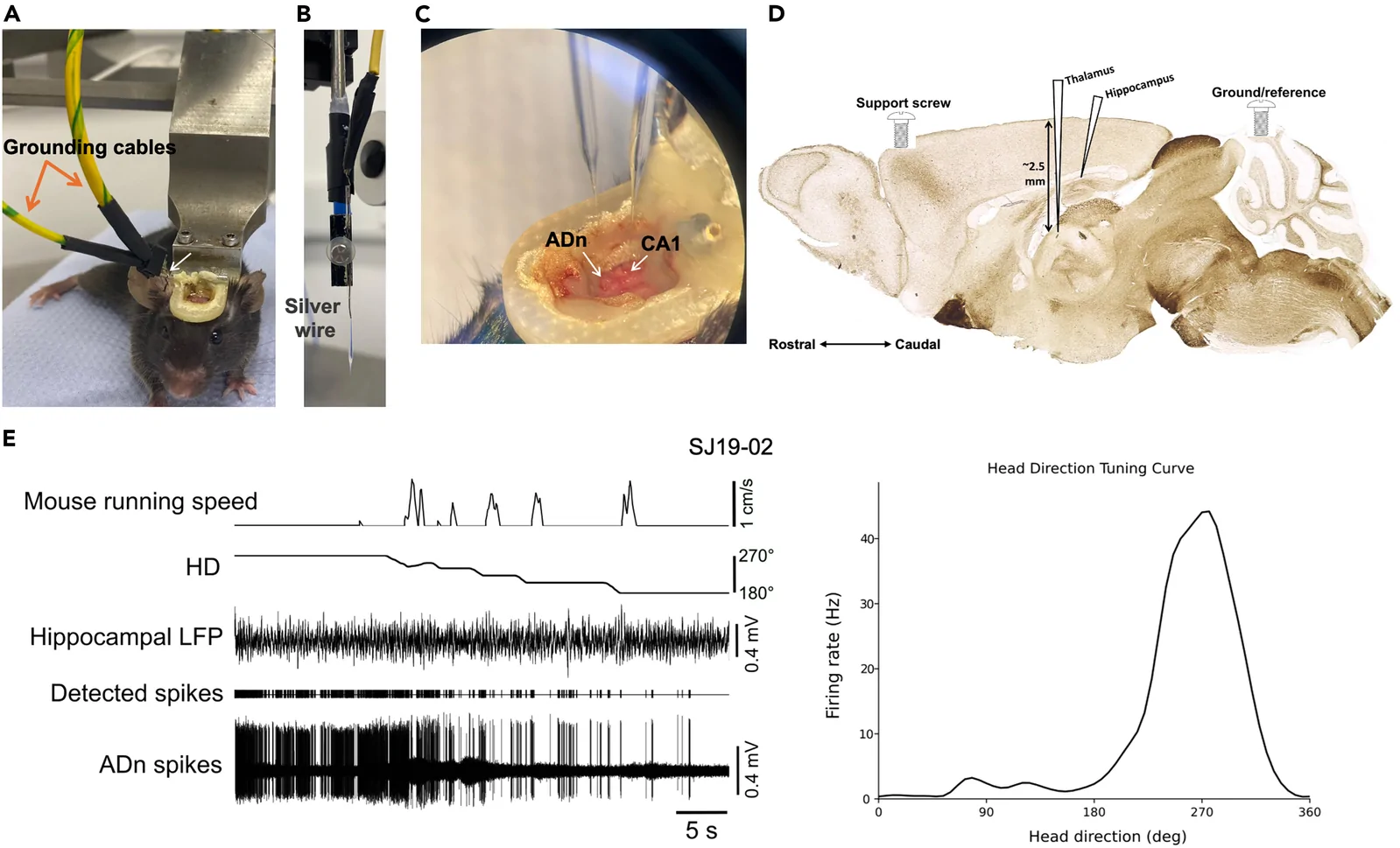
*In vivo* extracellular recordings and identification of ADn head direction cells (A) Head-fixed recording configuration showing the grounding cables (red arrows) connected to one of the implanted ground/reference pins (white arrow). (B) Glass recording pipette containing a chlorided silver wire that connects to the preamplifier. (C) Representative positioning of ADn and hippocampal CA1 glass electrodes above the craniotomy sites. (D) Sagittal brain section from Paxinos and Franklin^12^ (acetylcholinesterase staining) modified to illustrate the positions of two glass electrodes targeting the hippocampus and ADn, together with the locations of support and ground/reference screws. (E) Left: representative simultaneous recordings of mouse running speed, head direction (HD), hippocampal CA1 LFP, detected spikes, and neuronal activity (filtered spikes) of a single ADn HD cell (SJ19-02). Right: corresponding HD tuning curve of the recorded ADn neuron.
10. Carefully remove wax with sterile fine forceps to expose craniotomy sites then rinse with sterile saline.
11. Connect grounding cables from the preamplifiers to one of the ground/reference pins (Figure 3A).
12. Mount the glass pipette for CA1 onto the micromanipulator and insert the silver wire into the neurobiotin solution (Figure 3B).

***Note:*** Avoid introducing air bubbles or excessive pressure during insertion, as this may reduce recording stability.

8. Backfill glass pipettes with neurobiotin solution using a flexible needle. Ensure pipette tips are completely filled and free of air bubbles.
9. Head-restrain the mouse via the headplate (Figure 3A).

13. Position a pipette over one of the two CA1 craniotomy sites (Figure 1C).
  a. Lower the pipette to the brain surface under microscopic guidance.
  b. Add sterile saline to the craniotomy site.
  c. Test electrode impedance; the ideal range is 8–12 MΩ.
  d. Set the brain surface as the zero position of the z-axis of the micromanipulator.
14. Advance the pipette slowly (3–10 μm/s) using the motorized micromanipulator.
  a. Monitor signal quality using the data acquisition software.

***Note:*** CA1 is 1–1.1 mm dorsoventral (DV) from the brain surface (Figures 3C and 3D), identified based on theta oscillations during running periods and intermittent sharp-wave ripples during rest periods.^13^

15. Mount a glass pipette onto the other micromanipulator in order to target one of the two ADn craniotomy sites and repeat Steps 13–14 for the ADn-targeting pipette.

***Note:*** The ideal impedance for the ADn-targeting pipette is 11–15 MΩ, but even 30 MΩ is acceptable. The ADn is located 2.5–3.5 mm DV depending on the AP and ML position.

16. Periodically test electrode impedance while lowering the pipette to the target depth. Use a high frequency burst of current (‘buzz’) to clear debris from a blocked tip.

**CRITICAL:** Do not ‘buzz’ near the ADn target location as this can occasionally result in labeling of an unrecorded cell.

***Note:*** When lowering the pipette towards the ADn, carefully monitor the local field potential (LFP). The LFP has different spectral properties across the cortex, hippocampus, and thalamus. In the anterior thalamus, the mean amplitude is significantly lower compared to cortex and hippocampus. The ADn should be the first thalamic nucleus encountered if coordinates are accurate. The anterior ADn can be predicted based on noise in the LFP just above the target due to passing through the fimbria. The posterior ADn can be predicted based on spectral characteristics of CA3 and the dentate gyrus above the target, including prominent theta oscillations during movement, and sharp waves and dentate spikes during rest. Comparison to the CA1 LFP will facilitate this assessment (Figure 3E).

17. Record in the predicted ADn target region.
  a. Rotate the setup to change the animal’s head direction while adjusting the depth of the glass electrode in order to increase the chances of passing through receptive fields of HD cells.

***Note:*** ADn HD cells can be recognized through an audio monitor by the pitch of the action potentials (Audio S1), abrupt increases in firing frequency as the animal enters the preferred directional range, and non-rhythmic burst firing patterns within the receptive field (Figure 3E).

***Note:*** It is recommended to target the center of the craniotomy site over the ADn, and systematically adjust subsequent penetrations medio-laterally or antero-posteriorly if non-HD cells are encountered. Keep track of penetration positions relative to the craniotomy site, as this improves targeting efficiency over time.

18. Record HD cell activity (Figure 3E).
  a. Synchronize the IMU with the electrophysiological data acquisition software.
  b. Perform full clockwise and counterclockwise rotations at different speeds, including at least one slow full rotation suitable for subsequent analysis.
  c. Note down the cell name, penetration time, recording depth, and hemisphere.
19. If no HD cells are detected, slowly withdraw the pipette from the brain.
  a. Adjust the medio-lateral and/or antero-posterior position and/or craniotomy site.
  b. Repeat from Step 16.

***Note:*** Before starting a new penetration, note the previous penetration position and reassess impedance. A marked decrease in resistance may indicate a broken pipette tip, whereas excessively high resistance may reflect blockage. If either case happens, replace the pipette to improve recording conditions.

***Note:*** The glass pipette in CA1 typically does not need withdrawing when adjusting the position of the ADn-targeting pipette.

**CRITICAL:** Mechanical stability is essential for successful recordings and juxtacellular labeling. The air table should be operational to minimize vibrations. Animal movement, rapid rotation, or excessive manipulator adjustment can disrupt recording stability. Once stable HD tuning has been confirmed, it is often preferable to proceed immediately to juxtacellular labeling (Steps 20–22) rather than prolonging the recording, as stability can deteriorate within minutes. Small improvements in spike amplitude should be balanced against the increased risk of losing the recorded cell.

Audio S1. Action potentials from an ADn HD cell, related to Steps 17 and 18 Abrupt increases in firing are detected as the animal enters the preferred directional firing range of the HD cell.

### Juxtacellular labeling

**Timing: 5–20 min**

**Figure 4:**
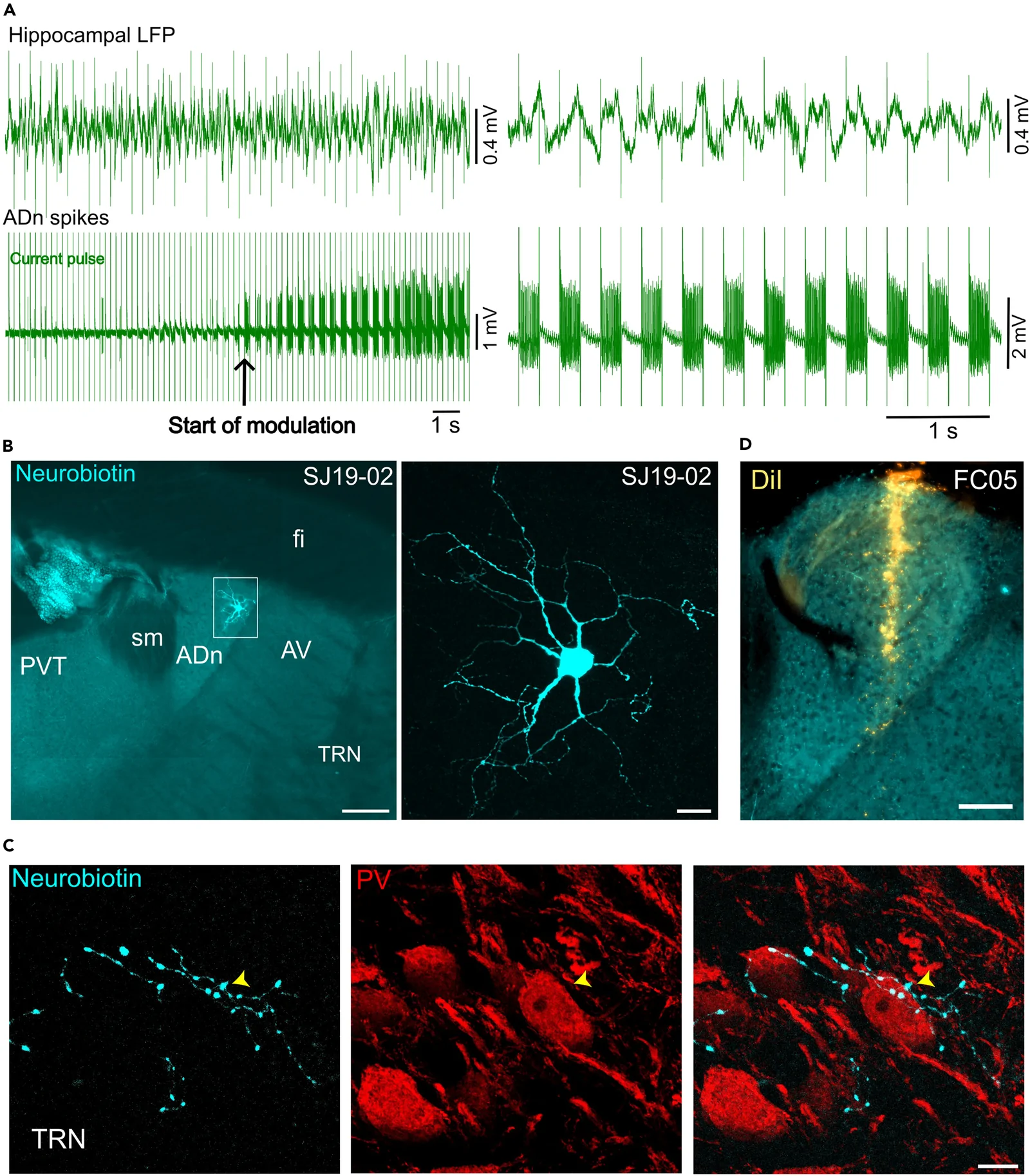
Juxtacellular labeling and anatomical recovery of head direction cells (A) Left: example of juxtacellular labeling. Before juxtacellular modulation, the recorded neuron typically displays spontaneous extracellular spikes with irregular timing and moderate amplitude. When current pulses are applied, spike traces appear as a shift from asynchronous events to rhythmic and synchronized firing aligned with the positive current pulses. Right: example of successful juxtacellular modulation. Spikes become tightly time-locked to injected current pulses while maintaining extracellular waveform characteristics. Regular vertical lines are artifacts of the current injection. (B) Left: epifluorescence micrograph of a coronal brain section showing a juxtacellularly labeled HD cell (neurobiotin, cyan, cell SJ19-02). Right: confocal maximum intensity z-projection (9 μm thick) showing the soma and dendrites of the HD cell. Scale bars: left, 200 μm; right, 20 μm. (C) Parvalbumin (PV) immunoreactive neurons (red) in the thalamic reticular nucleus (TRN) are innervated by boutons (cyan, e.g., arrowhead) arising from the axon of the HD cell shown in (B). Confocal maximum intensity z-projection (18.5 μm thick). Scale bar: 20 μm. (D) Recovery of a probe recording site (DiI fluorescence) from animal FC05. Scale bar: 100 μm. Abbreviations: AV, anteroventral thalamic nucleus; sm, stria medullaris; fi, fimbria; PVT, paraventricular thalamic nucleus.
  c. Continue modulation while monitoring spike entrainment and cell stability. Aim to modulate the cell for at least 3 minutes.

***Note:*** Modulation strength depends on several factors: distance between the pipette and the cell (indicated by spike amplitude – a larger spike amplitude increases the probability of cell modulation); modulation duration; pipette tip diameter (larger tips have a lower impedance); concentration of neurobiotin.

20. Once the cell has been characterized as an HD cell, slowly move the pipette closer to the cell, indicated by an increase in spike amplitude (to >1 mV).
  a. Deliver positive current pulses (200 ms on/200 ms off) in bridge mode, increasing the current gradually while dynamically adjusting the bridge balance.
  b. Listen for an increase in noise followed by an abrupt entrainment of action potential bursts encompassing the duration of each positive current pulse. This is known as modulation, which delivers neurobiotin into the cell. (Figure 4A)

21. Stop the current pulses, and withdraw the electrode slowly.

***Note:*** Spike amplitude should gradually decrease into the baseline noise, increasing the chances of *post-hoc* recovery; an abrupt burst would indicate cell death and limited chance of recovery.

22. After successful labeling, allow an incubation period of 4–6 h prior to transcardial perfusion to improve chances of recovering long-range axons.

***Note:*** At least 5 min of modulation at high amplitude should result in strong labeling of the soma, dendrites, and long-range axons including recovery of presynaptic terminals *post-hoc* (Figures 4B and 4C).

***Optional:*** Paint the glass pipette tip with lipophilic fluorescent tracers (e.g. DiI, DiO) before a penetration in order to visualize the pipette track *post hoc*.

### High-density probe recordings

**Timing: 30–120 min**

23. Optionally extend the experiment by performing targeted high-density recordings of HD cells in the same location as the juxtacellularly-labeled cell.
  a. After withdrawing the glass pipette, paint the tip with methylene blue solution.
  b. Lower the pipette to the brain surface to mark the exact recording site.
24. Paint a probe (e.g., 64-channel silicon probe) with a fluorescent tracer dye.
25. Exchange the glass pipette and preamplifier with the probe connected to a headstage and data acquisition board (e.g., Open Ephys).
26. Under microscopic guidance, position the tip of the probe at the methylene blue site on the brain surface.
  a. Apply additional sterile saline to the craniotomy sites.
27. Lower the probe to the target location (1 μm/s), monitoring the LFPs of each recording channel.
28. Stop at the target depth and leave probe to stabilize for a 2–3 minutes.
  a. Check for multiple unit activity.
  b. Synchronize the IMU with the data acquisition software.
  c. Perform clockwise and counterclockwise turns of the recording apparatus as in Step 18.
29. After recording, retract the probe slowly (2 μm/s).
30. Rinse the cranium with sterile saline and protect by completely covering with warm wax.

***Note:*** Steps 15–30 can be repeated for the ADn site in the other hemisphere.

### Identification of labeled HD cells

**Timing: 1–5 days**

31. Perfuse the animal.
  a. Deeply anesthetize the animal with pentobarbital (50 mg/kg, i.p.).
  b. Transcardially perfuse with saline followed by a fixative solution containing 4% paraformaldehyde, 15% v/v saturated picric acid, and 0.05% freshly-added glutaraldehyde (pH 7.3).
  c. Remove the brain and store in 0.1 M phosphate buffer (PB).

***Optional:*** If the brain is poorly fixed, postfix overnight with fixative lacking the glutaraldehyde.

32. Brain sectioning.
  a. Wash out excess fixative.
  b. Section the brain at 70 μm thickness using a vibrating microtome, collecting serial sections in 24-well plates.

***Note:*** Make a cut along the cortex of one hemisphere to facilitate matching recording locations to labeled cells.

33. Recover the neurobiotin-labeled cell (Figure 4B).
  a. Select sections containing brain regions of interest.
  b. Place sections in Tris-buffered saline with 0.3% Triton X-100 (TBS-Tx) containing Alexa Fluor 488-, Cy3-or Cy5-conjugated streptavidin for 4 h at 20–25°C or overnight at 4°C.
  c. Wash sections in PB.
  d. Mount sections to glass slides in mounting medium.
  e. Seal slides with nail varnish.

**CRITICAL:** If a fluorescent tracer (e.g. DiI) is present in the sections (Figure 4D), select streptavidin-conjugated fluorophores that do not spectrally overlap with the fluorescent tracer.

***Optional:*** If the labeled cell of interest is predicted to be strongly labeled, replace TBS-Tx with the freeze-thaw method of permeabilization for the majority of (or all) sections. In this case, cryoprotect sections in 20% sucrose in 0.1M PB for 4 h then subject to 2 rounds of rapid freezing and thawing over liquid nitrogen, followed by washing in PB, replacing TBS-Tx with PB for all steps.

34. Confirm location of recovered cells using a widefield fluorescence microscope (Figure 4B).
35. Determine the neurochemical profile of labeled HD cells and test synaptic targets using immunohistochemistry.
  a. Demount sections containing the neurobiotin-labeled processes.
  b. Incubate sections in primary antibody solution (3 d at 4°C) followed by 4 h or overnight at 4°C in secondary antibody solution.
  c. Remount sections and document with confocal microscopy (Figures 4B and 4C).

***Optional:*** Reconstruct labeled cells for neuronal morphology and projection patterns by processing serial sections with biotinylated horseradish peroxidase and avidin (‘ABC complex’) followed by 3,3′-diaminobenzidine (DAB). Freeze-thaw-processed sections are preferable due to restricted diffusion of the DAB reaction. Trace labeled dendrites and axons across serial sections using a light microscope with a drawing tube or use appropriate tracing software.

## Expected outcomes

Successful execution of this protocol is expected to yield single-cell recordings of HD cells in the ADn during passive rotation, with firing confined to a preferred directional range and comparable tuning across sessions. A large fraction (∼95%) of ADn neurons recorded with this method are expected to meet HD criteria (details about analysis of HD cells have been reported previously^1^). Recovered HD neurons after juxtacellular labeling are expected to be located in the ADn; their dendrites and long-range axons can be reconstructed.^1^ Combined with immunohistochemistry, the neurochemical identity of labeled cells as well as their synaptic target neurons can be determined (Figure 4).

## Limitations

Juxtacellular recordings are low-throughput and sensitive to movement artifacts. Candidate recorded cells can be lost before attempting to label. Passive rotation improves stability and minimizes artifacts, but does not fully reproduce all features of free behavior, and HD anchoring depends strongly on preserved visual cues. Recovery of long-range axons is variable and often benefits from longer recovery periods. Finally, success rates may differ across brain regions and also depends on experimenter experience.

## Troubleshooting

### Problem 1: The animal struggles during passive rotation (steps 5–7, 18, and 28)

Increase habituation duration and reduce rotation speed during early sessions. Maintain a calm cue-rich environment and avoid abrupt reversals.

### Problem 2: Stable single-cell recordings are difficult to maintain (steps 17–20)

Mechanical disturbance is a major cause of cell loss for awake juxtacellular recordings. Use new pipettes, reduce vibration, apply wax over craniotomy sites during recordings, and ensure micromanipulator adjustments are slow approaching (and within) the target depth.

### Problem 3: HD tuning is weak or unstable (step 18)

Check that angular tracking is synchronized, ensure the animal has access to stable visual landmarks, and verify that rotations cover the full directional range in both clockwise and counterclockwise directions. Ensure that cables are trailed behind the setup and are not caught on equipment or become trapped underneath the turntable. Exclude epochs with negligible rotation if using angular velocity thresholds in analysis.

### Problem 4: Juxtacellular labeling fails (steps 20–22)

Optimize current amplitude and pulse duration to achieve clear spike entrainment without losing the cell. If recovery of long-range axons is the main endpoint, allow 4–6 h survival before perfusion to ensure neurobiotin reaches distal presynaptic terminals. If a cell is damaged/injured during the labeling, either repeat the experiment the following day (neurobiotin will be metabolized and damaged cells will have been phagocytosed), or perfuse within 1 hour to recover a partially-labeled cell.

### Problem 5: Craniotomy sites cannot be visualized (steps 13–15)

In some experiments, especially for recording sessions that take place at least 3 days after the craniotomies, sites can become obscured due to growth tissue. Pipettes typically can penetrate this tissue without damaging the pipette tip. The zero z-position (brain surface) must be estimated. Attempt to conclude experiments before significant growth tissue appears, apply an anti-mitotic agent, and/or select another craniotomy site.

### Problem 6: The labeled cell cannot be assigned to a recording (steps 20–22 and 35)

In some cases, one labeling attempt can result in multiple labeled cells, which makes it difficult to match recordings to specific labeled cells. Possible reasons include cells connected via gap junctions, or the pipette is close to multiple somata/dendrites during juxtacellular modulation. For the latter, prior to the labeling attempt, if receptive fields of adjacent HD cells overlap it may be possible to detect lower amplitude spikes in addition to the main cell. Move the pipette as close as possible to the main cell (to obtain amplitudes > 1 mV) before the labeling attempt. In the case of multiple labeled neurons due to multiple attempts of labelling, if cell positions match the depths of recordings noted down and their fluorescence intensity matches the modulation durations, related recordings can be tentatively assigned based on whether labeled cells are within or outside the brain regions of interest; if not, these cells should be excluded from the dataset.

## Resource availability

### Lead contact

Further information and requests for resources and reagents should be directed to and will be fulfilled by the lead contact, Tim J. Viney (tim.viney@pharm.ox.ac.uk).

### Technical contact

Technical questions on executing this protocol should be directed to and will be answered by the technical contact, Tim J. Viney (tim.viney@pharm.ox.ac.uk).

### Materials availability

This study did not generate new unique reagents.

### Data and code availability

• Electrophysiological data have been deposited at https://doi.org/10.5281/zenodo.20124105.^14^
• Code can be found at https://github.com/hasselmonians/Jiang_Hijazi_2025^15^ and https://github.com/neuralcircuitsTV/spikes_juxta.^16^
• Any additional information required to reanalyze the data reported in this paper is available from the lead contact upon request.

## Acknowledgments

We acknowledge support from the 10.13039/501100017506Alzheimer’s Society grant 522 AS-PhD-19a-010 (T.J.V.), 10.13039/100031707Medical Research Council grants MR/R011567/1 and MR/Z504518/1 (T.J.V.), the 10.13039/501100004789John Fell Fund grant 0013781 (T.J.V.), 10.13039/100014013UKRI grant EP/Z001358/1 (T.J.V. and S.H.), 10.13039/501100002283Alzheimer’s Research UK ARUK-RF2023B-026 (S.H.), and the 10.13039/501100000319ARUK Thames Valley Small Equipment Award AVR01965 (S.H.). S.H. was supported by a Blaschko Fellowship from the Department of Pharmacology, Oxford. S.J. was supported by a Clarendon Scholarship. We thank Prof. Claudia Gargini and Prof. Ilaria Piano from the Department of Pharmacy of the University of Pisa for enabling and supporting F.C. research stay in Oxford.

## Author contributions

S.J., S.H., and T.J.V. conceived and designed the project. S.J., F.C., S.H., and T.J.V. performed experiments. S.J., F.C., and S.H. analyzed data. S.J. and F.C. wrote the initial manuscript and made the figures. All authors edited the manuscript and approved the final version.

## Declaration of interests

The authors declare no competing interests.
